# Exploration of mRNAs and miRNA classifiers for various ATLL cancer subtypes using machine learning

**DOI:** 10.1186/s12885-022-09540-1

**Published:** 2022-04-21

**Authors:** Mohadeseh Zarei Ghobadi, Rahman Emamzadeh, Elaheh Afsaneh

**Affiliations:** 1 Department of Cell and Molecular Biology and Microbiology, Faculty of Biological Science and Technology, University of Isfahan, Isfahan, Iran; 2 Department of Physics, University of Isfahan, Hezar Jarib, Isfahan, 81746 Iran

**Keywords:** HTLV-1, ATLL, Asymptomatic carriers, Machine learning, ATLL subtypes

## Abstract

**Background:**

Adult T-cell Leukemia/Lymphoma (ATLL) is a cancer disease that is developed due to the infection by human T-cell leukemia virus type 1. It can be classified into four main subtypes including, acute, chronic, smoldering, and lymphoma. Despite the clinical manifestations, there are no reliable diagnostic biomarkers for the classification of these subtypes.

**Methods:**

Herein, we employed a machine learning approach, namely, Support Vector Machine-Recursive Feature Elimination with Cross-Validation (SVM-RFECV) to classify the different ATLL subtypes from Asymptomatic Carriers (ACs). The expression values of multiple mRNAs and miRNAs were used as the features. Afterward, the reliable miRNA-mRNA interactions for each subtype were identified through exploring the experimentally validated-target genes of miRNAs.

**Results:**

The results revealed that miR-21 and its interactions with DAAM1 and E2F2 in acute, SMAD7 in chronic, MYEF2 and PARP1 in smoldering subtypes could significantly classify the diverse subtypes.

**Conclusions:**

Considering the high accuracy of the constructed model, the identified mRNAs and miRNA are proposed as the potential therapeutic targets and the prognostic biomarkers for various ATLL subtypes.

**Supplementary Information:**

The online version contains supplementary material available at 10.1186/s12885-022-09540-1.

## Background

Adult T-Cell Leukaemia/Lymphoma (ATLL) is a type of cancer disease which is developed due to the infection by Human T-Cell Leukemia Virus type 1 (HTLV-1). It provides the aggressive malignant of CD4+ T lymphocytes [1]. In fact, the infection by HTLV-1 can lead to the progression of two main diseases including ATLL and HTLV-1-Associated Myelopathy/Tropical Spastic Paraparesis (HAM/TSP).

HTLV-1 is an endemic virus with the prevalence of more than 20 million people worldwide in several regions, including, the East North of Iran, some parts of South America, the Caribbean, and Japan. ATLL develops in about 5% of the infected patients after a long dormancy period which are called Asymptomatic Carriers (ACs) [2].

Two main viral proteins are the viral transactivating protein Tax-1 and HTLV-1 bZIP factor / HTLV-1 basic-zipper factor (HBZ) which have critical roles in the development of diseases. Tax-1 implicates the transformation and the proliferation of the infected T cells. However, ATLL cells often lose the Tax expression because of the epigenetic and genetic alterations in the proviral genome. Furthermore, HBZ protects the proliferation of ATLL cells [3, 4].

ATLL is categorized into four main subtypes according to Shimoyama classification: acute, chronic, smoldering, and lymphoma [5, 6]. The acute and lymphoma subtypes are characterized by aggressive behavior and poor prognosis. While the chronic and smoldering subtypes are specified by an indolent clinical course and different clinicopathologic features. The hepatosplenomegaly and elevated lactate dehydrogenase are observed in the acute type and also less frequently in the lymphoma type [7]. In addition, the acute type is identified by unusual lymphocytes in the peripheral blood and the blood circulating. The chronic subtype usually causes leukocytosis with absolute lymphocytosis, skin rash, hypercalcemia, and moderate lymphadenopathy [8, 9]. The smoldering subtype is asymptomatic which is specified by less than 5% circulating irregular lymphoid cells without organomegaly or hypercalcemia [10].

Several studies explored the possible pathogenesis mechanisms of the HTLV-1 infection in ACs toward ATLL and/or HAM/TSP [2, 11–15]. However, some of them considered ATLL disregarding the subtypes. In addition, the subtypes of ATLL have poor prognosis due to the inherent chemoresistance and the intense immunosuppression. Moreover, the manifestations and cycles of the disease are heterogeneous [16]. Therefore, for identifying the subtypes of ATLL with the highest accuracy and also for selecting the conventional treatments, the computational classification methods could be beneficial.

In this investigation, we utilized a machine learning method for classifying three subtypes of ATLL. It led to finding the powerful mRNAs and miRNA classifiers between these subtypes and ACs. The identified classifiers could determine the pathogenesis routes from the infected HTLV-1 toward the development of each ATLL subtype.

## Materials and methods

### Dataset collection and preprocessing

We downloaded four microarray datasets, from the Gene Expression Omnibus (GEO) repository website. The datasets including GSE55851 [17] and GSE33615 [18] contain the genes expression in the whole blood or the Peripheral Blood Mononuclear Cells (PBMCs) of three subtypes including acute, chronic, and smoldering.

The GSE29332 [19] and GSE29312 [19] include the gene expression in the PBMCs of AC carriers. A total of 29 acute, 23 chronic, and 10 smoldering ATLL subjects, as well as 37 ACs samples containing 15,565 common genes, were used for further analysis. Moreover, to find the miRNA classifiers, the datasets were employed with the accession numbers GSE46345 [20] and GSE31629 [18]. They contain the miRNA expressions of ACs and ATLL subjects. A total of 12 ACs and 40 ATLL samples including the expression of 549 miRNAs were involved in the analysis. The characteristics of the datasets are specified in Table 1. To remove the batch effect among the datasets, the function of removeBatchEffect in the Limma package was employed [21]. The data were randomly divided into the train and test sets in Python (65/35).

**Table 1:** Characteristics of datasets included in the analysis

**Dataset**	**ACs**	**Number of Samples**
GSE29312	Illumina HumanHT-12 V3.0 expression beadchip	ACs: 20
GSE29332	Illumina HumanWG-6 v3.0 expression beadchip	ACs: 17
GSE46345	Agilent-021827 Human miRNA Microarray (V3)	ACs: 12
	**ATLL**	
GSE33615	Agilent-014850 Whole Human Genome Microarray 4x44K G4112F	Acute: 26 Chronic: 20 Smouldering: 4
GSE55851	Agilent-026652 Whole Human Genome Microarray 4x44K v2	Acute: 3 Chronic: 3 Smouldering: 6
GSE31629	Agilent-019118 Human miRNA Microarray 2.0 G4470B	ATLL: 40

### Support vector machine-recursive feature elimination with cross-validation (SVM-RFECV)

Here, to determine the specific features that can classify the various ATLL subtypes, SVM-RFECV based on the tenfold cross-validation was employed [22]. RFE is a wrapper variable selection approach that utilizes the interior filter-based variable selection. SVM-RFE is principally a backward elimination manner, in which the top-ranked features are the most relevant conditional variables on the special ranked subset in the model. The top-ranked features in the final iteration of SVM-RFE are the substantial informative variables and the bottom-ranked features are the insubstantial ones that can be removed [23]. SVM-RFECV comprises five steps: 1) Training the train set by the tenfold cross-validation SVM; 2) Ordering the variables using the weights of the obtained classifier; 3) Eliminating the variables with the smallest weight; 4) Updating the training dataset according to the chosen variables; 5) Repeating the steps with the training set limited to the remaining variables [24]. We employed SVM-RFECV algorithm in Python 3.9.

### Identification of differentially expressed genes (DEGs)

To determine differentially expressed genes between each ATLL subtype and the AC samples, the Limma package in R environment programming was employed [25]. Benjamini-Hochberg FDR adjusted *p*-values < 0.05 and logFC = |5| were chosen as the criteria for exploring the remarkable DEGs.

### Determination of target genes of miRNAs

To find the experimentally validated target genes of miRNAs, miRTarBase database [15, 26] was used. The network of miRNA-target genes was visualized by Cytoscape 3.6.1.

### Pathway enrichment analysis

In order to pathway enrichment analysis of the identified classifier genes for each subtype, the ToppGene database was employed [27]. The terms with adj.P.value < 0.05 were determined as statistically remarkable.

## Results

### Determination of DEGs

**Fig. 1:**
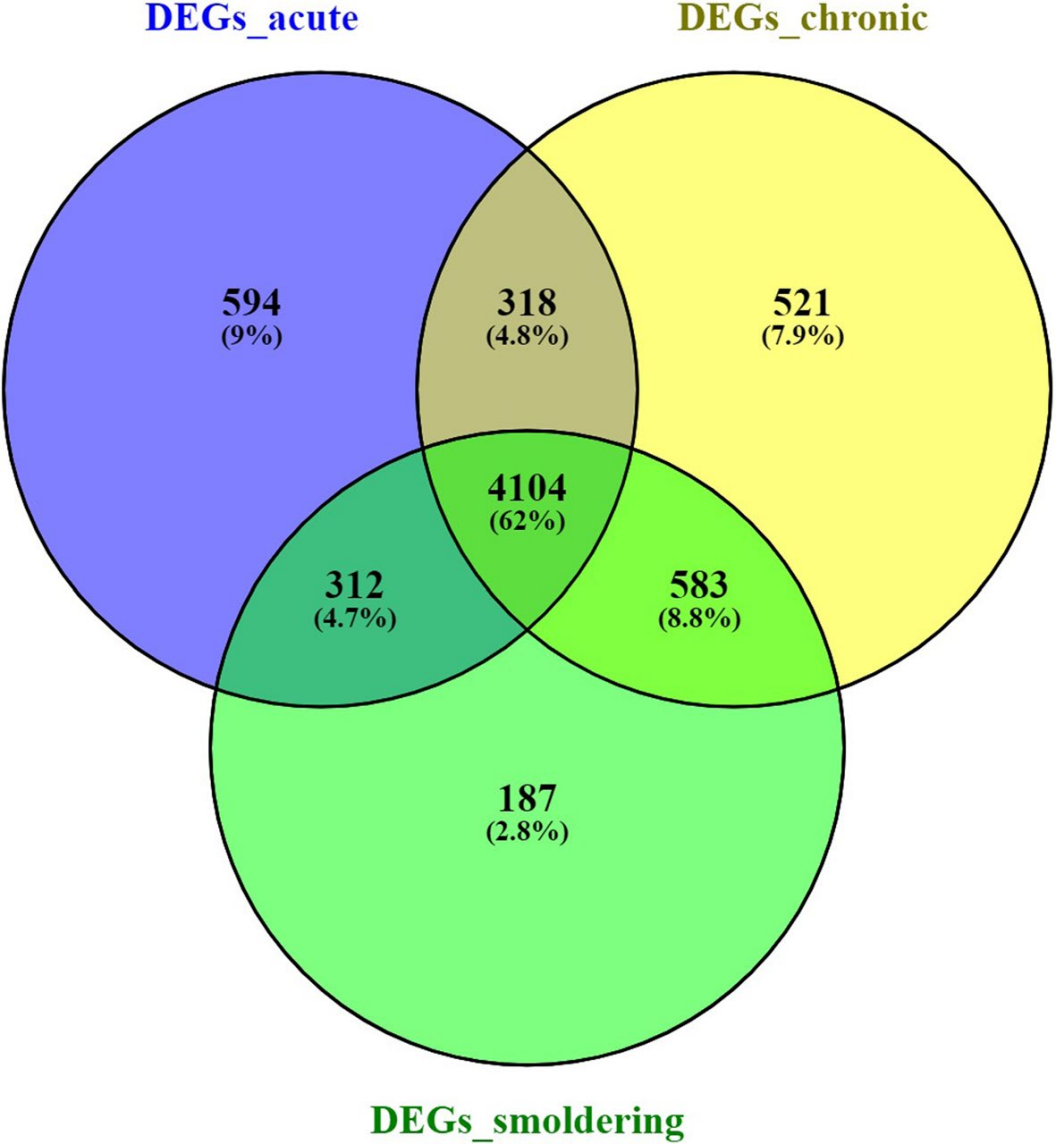
Venn diagram containing DEGs of acute, chronic, and smoldering ATLL subtypes

A total of 5327, 5525, and 5185 DEGs were found among ACs with ATLL_acute, ATLL_chronic, and ATLL_smoldering, respectively (Supplementary data file 1). Afterward, the unique DEGs belonging to each subtype were explored. The Venn diagram shows 521, 594, and 187 unique DEGs for ATLL_chronic, ATLL_acute, and ATLL_smoldering, respectively (Fig. 1). These DEGs were considered the selected variables for each subtype (Supplementary data file 2). Therefore, the matrices containing the expression values of the selected features for each sample were constructed for machine learning.

### Classification of ATLL subtypes using SVM-RFECV

**Fig. 2:**
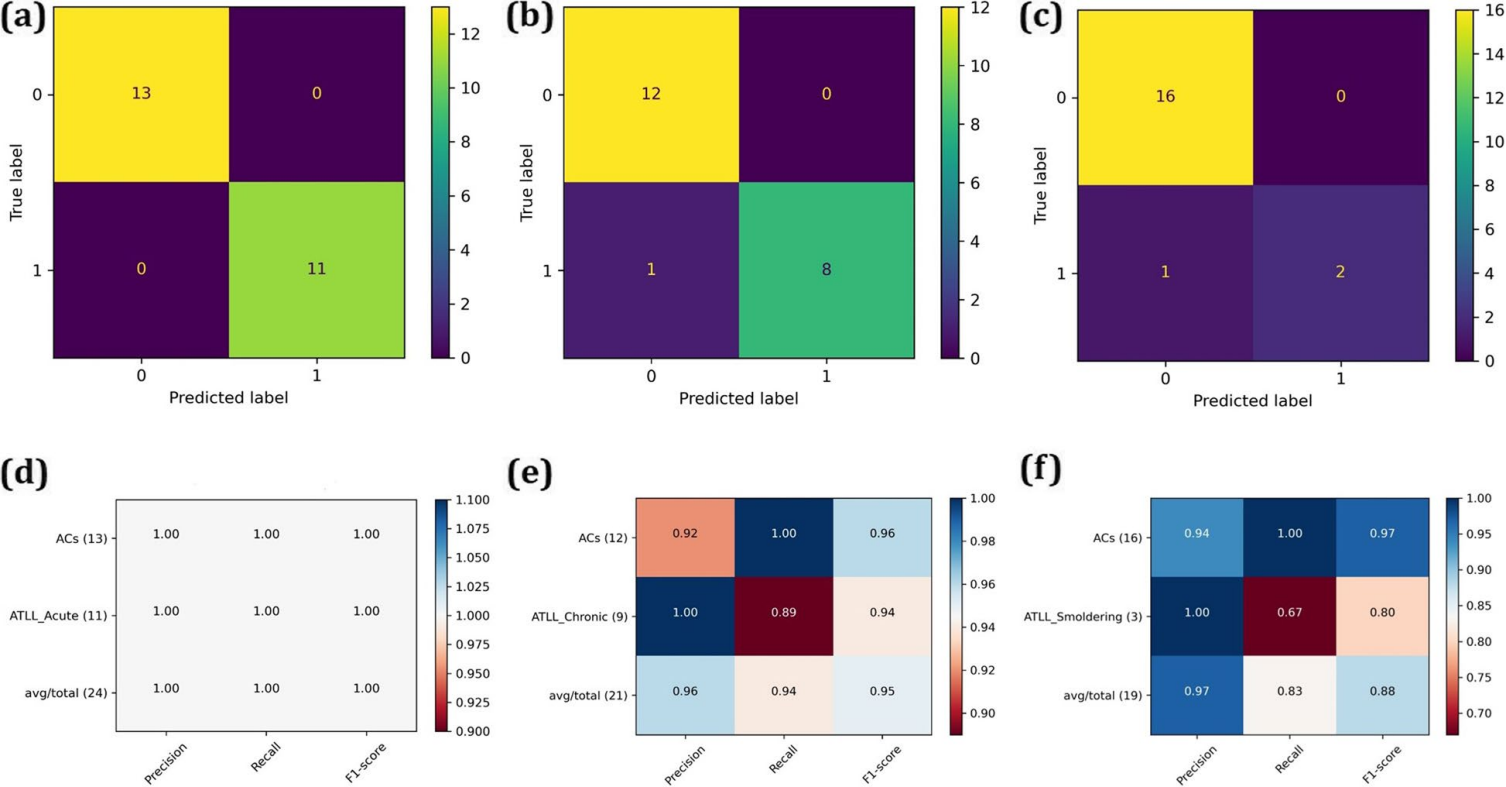
The confusion matrix (**a-c**) and classification reports (**d-f**) for ATLL_acute, ATLL_chronic, and ATLL_smoldering subtypes

The SVM-RFECV analysis was utilized to find the features that could classify the various ATLL subtypes from ACs. For this purpose, unique DEGs for each subtype were used in the train data. To validate the SVM model, the test sets were under-investigated. The accuracy results and the selected features are mentioned in Table 2. A total of 27, 9, and 32 genes were found as the best classifiers for ATLL_acute, ATLL_chronic, and ATLL_smoldering, respectively. Furthermore, the confusion matrix and the classification reports for the test sets are visualized in Fig. 2a-f. The results showed that the selected features could significantly classify the various subtypes of ACs. The accuracy for the test set was found as 1.00, 0.95, and 0.95 for the ATLL_acute, ATLL_chronic, and ATLL_smoldering, respectively. In order to find the activated pathways by the genes classifiers for each subtype, the pathway enrichment analysis was performed. The involvement of each gene in each pathway and also the previously reported function of the genes in the ATLL progression were mentioned in Supplementary data file 3.

**Table 2:** List of selected features and accuracy of model

Results	ATLL_acute	ATLL_chronic	ATLL_smouldering
Subtypes			
**Features**	IDH2,PTGER3,TM2D2,DAAM1,MXD1,RALB,TSC22D4,FRY,NRSN2,SPINK2,GBP3,PAPSS1,SRM,HYI,PDIA4,STON1,E2F2,NDST2,RNF35,UBQLN1,FHL2,NDUFAF1,SLC39A11,WDR41,FLVCR1,NINJ2,SMS,XAF1	CD40LG,MAP1LC3C,SMAD7,PUS1,RORC,ADAMTS10,TRMT61A,CCT5,VCL	CDCA7L,HSPA1A,MCAT,SLC25A21,CHN1,IFI44,MT1G,SLC6A20,CSRNP1,INPP5F,MYEF2,STMN1,NCF2,NOSIP,CCDC50,ENO3,LAG3,RELA,WWC3,CCL3,FOSL2,LSR,RNASEH2C,BHLHE40,DUSP23,KCNH5,PARP1,TTN,CD70,HOXB2,MAF,SAP30
**Accuracy**	0.975 (0.075)	0.942 (0.118)	1.00 (0.00)

The genes classifiers for ATLL_acute were enriched in Glutathione metabolism, Urea cycle and the metabolism of amino groups, beta-Alanine metabolism, Cysteine and methionine metabolism, sulfate activation for sulfonation, CXCR4-mediated signaling events, Metabolism of polyamines, Amino Acid metabolism, Metabolic pathways, Pathways in cancer, Hypoxia and p53 in the Cardiovascular system, Interferon Signaling, the planar cell polarity Wnt signaling, Noncanonical Wnt signaling pathway, Expression of cyclins regulates progression through the cell cycle by activating cyclin-dependent kinases.

In addition, the genes classifiers for ATLL_chronic in tRNA modification in the nucleus and cytosol, TGF-beta Receptor Signalling in Skeletal Dysplasias, tRNA processing, altered transforming growth factor-beta Smad dependent signaling, Cell to Cell Adhesion Signaling, CD40L Signaling Pathway, Cytokine Signaling in Immune system, Hypoxia response via HIF activation, Primary immunodeficiency, MAP2K and MAPK activation, IFN-gamma pathway, Integrins in angiogenesis, TGF-beta receptor signaling, IL4-mediated signaling events, Signaling events mediated by VEGFR1 and VEGFR2, Signaling by Interleukins, Non-genomic actions of 1,25 dihydroxy vitamin D3, Oncogenic MAPK signaling, Ferroptosis, Folding of actin by CCT/TriC.

For ATLL_smoldering, the classifiers were enriched in IL-18 signaling pathway, Chaperones modulate interferon Signaling Pathway, Rac 1 cell motility signaling pathway, NAD Metabolism in Oncogene-Induced Senescence and Mitochondrial Dysfunction-Associated Senescence, fMLP induced chemokine gene expression in HMC-1 cells, Osteoclast differentiation, CAMKK2 Pathway, RAC1/PAK1/p38/MMP2 Pathway, MAPK Signaling Pathway, Th1 and Th2 cell differentiation, NF-kappa B signaling pathway, MAPK signaling pathway, HIF-1 signaling pathway, Toll-like receptor signaling pathway, Acetylation and Deacetylation of RelA in The Nucleus, Apoptosis, NAD+ metabolism, Apoptotic Signaling in Response to DNA Damage, Downregulation of SMAD2/3:SMAD4 transcriptional activity, Fatty acid biosynthesis, D4-GDI Signaling Pathway, Metallothioneins bind metals, NRF2 pathway, 3-phosphoinositide degradation, TFs Regulate miRNAs related to cardiac hypertrophy, Metabolism of nitric oxide, VLDL interactions, Pathways of nucleic acid metabolism and innate immune sensing, Circadian rhythm pathway, Transcriptional misregulation in cancer, Signaling events mediated by HDAC Class I.

### Finding miRNA-gene classifier between ATLL subtypes and ACs

**Fig. 3:**
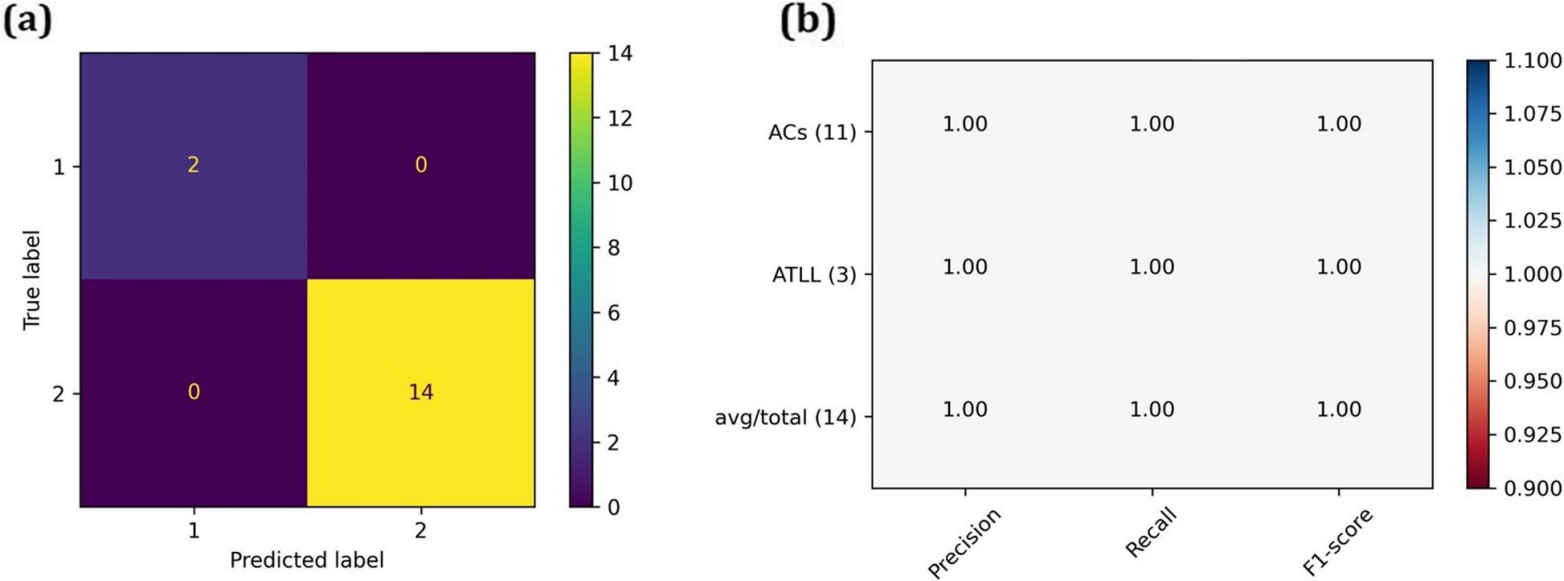
The (**a**) confusion matrix and (**b**) classification reports for ATLL_miRNA

**Fig. 4:**
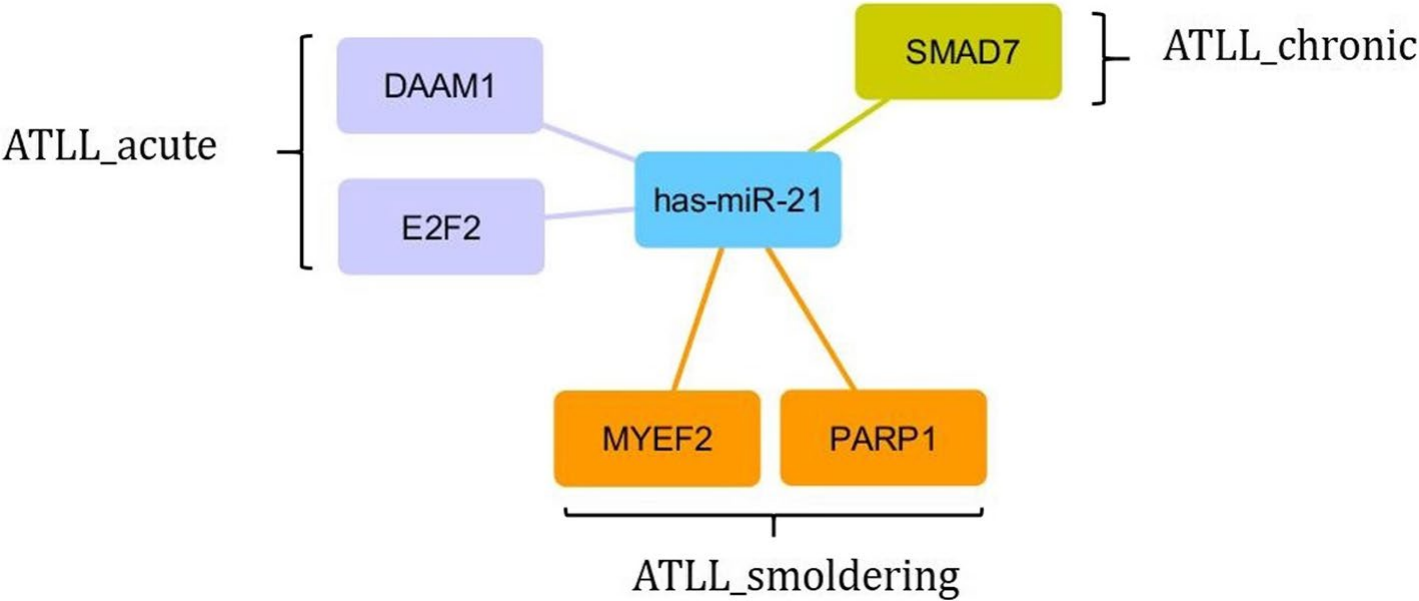
The miR-21-gene target interaction for various ATLL subtypes

As there are no reliable datasets to investigate the miRNA expression through ATLL subtypes, we considered miRNA expression in ATLL, generally. The SVM_RFECV analysis revealed the miR-21 as the best miRNA with an accuracy of 100% for classifying the ATLL from ACs. The confusion matrix and classification report are depicted in Fig. 3a, b. The target genes of this miR-21 were then found in the miRTarBase database (Supplementary data file 4). Next, the common genes were identified between the target genes and the classifier ones in each subtype. As a result, DAAM1 and E2F2 in acute, SMAD7 in chronic, MYEF2 and PARP1 in smoldering subtypes were specified (Fig. 4).

## Discussion

ATLL cancer is considered one of the extremely aggressive T cell non-Hodgkin lymphoma variants. Four clinical variants of ATLL have been specified: acute, lymphoma-type (lymphomatous), chronic, and smoldering. Shimoyama’s criterion is limited for classifying some patients in the lack of a purposeful immunophenotypic precisely and clonal analysis of peripheral blood [28]. For example, HTLV-1 carriers without ATLL can contain up to 5% of blood-circulating atypical cells, which causes clinicians to classify the lymphomatous ATLL with circulating atypical cells as acute. Moreover, it has been reported that ATLL patients in different regions respond differently to accessible therapies. For instance, first-line zidovudine interferon-α (AZT-IFN) can be beneficial for the aggressive leukemic ATLL patients in the United States [28]. Moreover, AZT-IFN is a first-line choice for patients with non-bulky aggressive ATLL and non-lymphomatous. It can also be the best election for the patients with chronic-type ATLL. On the other hand, chemotherapy is a preferred option for the lymphomatous. It is the favored etoposide-based regimen for patients with aggressive ATLL in Latin America. While AZT-IFN is a well first-line choice for the acute subtype [29].

A recent study on Japanese patients disclosed the unsatisfactory prognosis of the acute ATLL type and the worse prognosis of the smoldering type [30]. As a result, the accurate classification of ATLL subtypes could be applied for the proper treatments. ATLL subtypes could be categorized into molecularly distinguished subsets with various prognoses. Moreover, genetic profiling could contribute to obtain the better management and prognostication of ATLL patients [31]. Each ATLL subtype can carry diverse genomic alterations and different clinical courses. In a recent study, the total structural variations, mutations, driver alterations, and abnormal CN segments were explored in the aggressive (acute) and the indolent (chronic and smoldering) subtypes [32]. In this study, we concentrate on the expression values of coding and non-coding RNAs. We applied the support vector machine-recursive feature elimination as a machine learning approach to classify the ATLL subtypes from ACs samples. Then, we identified the potential prognostic targets.

Acute ATLL includes the lymphoma cells that persist in the blood. The main characteristic of this subtype is its aggressive biology, with a median survival of only 4–6 months. The disease progresses rapidly in the bones, skin, lymph nodes, spleen, and liver. *DAAM1* and E2F2 are two specific classifier genes for the acute ATLL. *DAAM1* encodes a protein that contains two FH domains pertaining to the FH protein subfamily with a role in the cell polarity. It is likely acts as a scaffolding protein for the Wnt-induced assembly of a disheveled (Dvl)-Rho complex. It also boosts the nucleation and elongation of the new actin filaments and regulates the cell growth by the microtubules’ stabilization. Moreover, it has been shown that DAAM1 can help the migration and the invasion of cancerous cells. Also, it can promote tumor advancement in Hepatocellular Carcinoma as well as breast and ovarian cancers [33–35].

The E2F2 protein is a transcription factor that has a substantial function in controlling the action of the tumor suppressor proteins and the cell cycle. Also, it is considered a target for the transforming proteins of the small DNA tumor viruses [36]. Particularly, E2F2 binds to the RB1 in a cell-cycle-dependent manner. RB1 mediates the control of the cell cycle through binding the E2F2 and also suppressing the expression from the E2F2-dependent promoters. It is concluded that E2F2 and DAAM1 could be considered for the prognosis of the acute ATLL subtype.

Another subtype of ATLL is chronic which is characterized by slow growth with an effect on the lungs, skin, lymph nodes, spleen, and liver. A higher number of T cells and lymphocytes in the blood are the signs of this subtype. SMAD7 encodes a nuclear protein that binds the E3 ubiquitin ligase SMURF2. After binding, this complex translocates to the cytoplasm and it can interact with TGFBR1 which results in the degradation of both the encoded protein and TGFBR1. The relationship between the expression of SMAD7 and lymphatic metastasis in gastric cancer has been reported [37]. Moreover, the survival of cancer cells and apoptosis were induced after SMAD7 transduction. The upregulation of SMAD7 interdicts the proliferation, boosts apoptosis, and inactivates the Smad signaling [38].

Smoldering ATLL similar to the chronic subtype grows slowly and affects the lungs or skin. It causes unusual T cell counts in the blood. *MYEF2* and *PARP1* are two classifier genes that we identified for the smoldering subtype. *MYEF2* is the myelin expression factor 2, which acts as a transcription suppressor of the myelin basic protein (MBP). MYEF2 is a downstream target that is modulated by the Wnt/β-catenin pathway. The genes regulated by Wnt/β-catenin can help for identifying the pathogenesis mechanisms of cancer and therapies [39]. Furthermore, the possible carcinogenesis role of MYEF2 has been proposed; however, its performance in cancer is still unknown and it should be evaluated in further studies.

*PARP1* encodes a chromatin-associated enzyme, namely, poly (ADP-ribosyl) transferase, which rectifies several nuclear proteins by poly (ADP-ribosyl)ation. The modification relies on DNA and is implicated in the regulation of different significant cellular processes like the proliferation and the transformation of the tumor. Also, the regulation of the molecular events is involved in the cell recovery from DNA damage [40].

PARP1 is a coactivator for the HTLV-1 transcription activator Tax. It constitutes the active complexes on the promoter [41]. Furthermore, the expression of PARP1 is related to a progressive course of indolent mantle cell lymphoma. Therefore, it was proposed that PARP1 could be used for the initial diagnostic studies as a negative predictor [42].

Moreover, SVM-RFECV was employed for finding a promising classifier of miRNA. MiR-21 was identified as the best classifier between ATLL and ACs. It involves the acceleration of tumorigenesis and the onset of some tumor types [43]. It can target many genes as well as the above-mentioned genes which are involved in the progression of cancer and tumor. Therefore, its function should be surveyed in a complicated network of genes and the effect of other miRNAs.

Our study has some limitations. It is known that the chronic type is divided into favorable and unfavorable types based on some laboratory findings. The unfavorable chronic type is regarded as aggressive ATLL as well as the acute type. There are no expression data regarding these two groups, so we had to consider chronic ATLL generally regardless of subgrouping. Moreover, the identified classifiers should be experimentally validated in a large cohort containing the samples from various ATLL subtypes.

## Conclusion

In summary, we identified the mRNAs and miRNA classifiers which could accurately classify the various ATLL subtypes vs. ACs. The outcomes disclosed the promising classifiers: SMAD7 in chronic, both MYEF2 and PARP1 in smoldering, and also both DAAM1 and E2F2 in acute subtypes. Moreover, miR-21 classified ATLL from ACs. However, further studies should be carried out to assess these classifiers, experimentally.

## Supplementary Information

**Additional file 1: Supplementary data file 1.** List of DEGs for each ATLL subtype.

**Additional file 2: Supplementary data file 2.** List of unique DEGs for each ATLL subtype.

**Additional file 3: Supplementary data file 3.** The involvement of each gene in each pathway and the previously reported function of genes in the ATLL progression.

**Additional file 4: Supplementary data file 4.** The target genes of miR-21.

## Data Availability

All data generated or analyzed during this study are included in this published article [and its supplementary information files].

## Abbreviations

ATLL: Adult T-Cell Leukaemia/Lymphoma
HTLV-1: Human T-Cell Leukemia Virus Type 1
ACs: Asymptomatic Carriers
SVM: Support Vector Machine
RFE: Recursive Feature Elimination
DEGs: Differentially Expressed Genes
SVM-RFECV: Support Vector Machine-Recursive Feature Elimination with Cross-Validation
HAM/TSP: HTLV-1-Associated Myelopathy/Tropical Spastic Paraparesis

## References

[1] Takatsuki K, Yamaguchi K, Kawano F, Hattori T, Nishimura H, Tsuda H, Sanada I, Nakada K, Itai Y (1985). Clinical diversity in adult T-cell leukemia-lymphoma. Cancer Res.

[2] Zarei Ghobadi M, Emamzadeh R, Teymoori-Rad M, Mozhgani S-H (2021). Decoding pathogenesis factors involved in the progression of ATLL or HAM/TSP after infection by HTLV-1 through a systems virology study. Virol J.

[3] Nakahata S, Ichikawa T, Maneesaay P, Saito Y, Nagai K, Tamura T, Manachai N, Yamakawa N, Hamasaki M, Kitabayashi I (2014). Loss of NDRG2 expression activates PI3K-AKT signalling via PTEN phosphorylation in ATLL and other cancers. Nat Commun.

[4] Ji Y, Matsuoka M (2007). Leukaemogenic mechanism of human T-cell leukaemia virus type I. Rev Med Virol.

[5] Oshiro A, Tagawa H, Ohshima K, Karube K, Uike N, Tashiro Y, Utsunomiya A, Masuda M, Takasu N, Nakamura S (2006). Identification of subtype-specific genomic alterations in aggressive adult T-cell leukemia/lymphoma. Blood.

[6] Shimoyama M. Adult T-cell leukemia/lymphoma and its clinical subtypes from the viewpoints of viral etiology. In: Human T-Cell Leukemia Virus. Berlin: Springer; 1985. p. 113–25. 10.1007/978-3-642-70113-9_8 2983939

[7] Kikuchi M, Jaffe ES, Ralfkiaer E . Adult T cell leukaemia/lymphoma. In: Jaffe ES, Harris NL, Stein H, Vardiman JW, editors. Pathology and Genetics of Tumours of Haematopoietic and Lymphoid Tissues. World Health Organization Classification of Tumours. Lyon: IARC Press; 2001. p. 200–203.

[8] Matutes E (2007). Adult T-cell leukaemia/lymphoma. J Clin Pathol.

[9] Qayyum S, Choi JK (2014). Adult T-cell leukemia/lymphoma. Arch Pathol Lab Med.

[10] Jabbour M, Tuncer H, Castillo J, Butera J, Roy T, Pojani J, Al-Malki M, Al-Homsi A (2011). Hematopoietic SCT for adult T-cell leukemia/lymphoma: a review. Bone Marrow Transplant.

[11] Zarei Ghobadi M, Mozhgani S-H, Erfani Y (2021). Identification of dysregulated pathways underlying HTLV-1-associated myelopathy/tropical spastic paraparesis through co-expression network analysis. J Neurovirol.

[12] Mozhgani S-H, Piran M, Zarei-Ghobadi M, Jafari M, Jazayeri S-M, Mokhtari-Azad T, Teymoori-Rad M, Valizadeh N, Farajifard H, Mirzaie M (2019). An insight to HTLV-1-associated myelopathy/tropical spastic paraparesis (HAM/TSP) pathogenesis; evidence from high-throughput data integration and meta-analysis. Retrovirology.

[13] Mozhgani SH, Zarei-Ghobadi M, Teymoori-Rad M, Mokhtari-Azad T, Mirzaie M, Sheikhi M, Jazayeri SM, Shahbahrami R, Ghourchian H, Jafari M (2018). Human T-lymphotropic virus 1 (HTLV-1) pathogenesis: a systems virology study. J Cell Biochem.

[14] Zarei Ghobadi M, Emamzadeh R (2022). Integration of gene co-expression analysis and multi-class SVM specifies the functional players involved in determining the fate of HTLV-1 infection toward the development of cancer (ATLL) or neurological disorder (HAM/TSP). PLoS One.

[15] Ghobadi MZ, Emamzadeh R, Mozhgani S-H (2021). Deciphering microRNA-mRNA regulatory network in adult T-cell leukemia/lymphoma; the battle between oncogenes and anti-oncogenes. PLoS One.

[16] Hermine O (2015). ATL treatment: is it time to change?. Blood.

[17] Fujikawa D, Nakagawa S, Hori M, Kurokawa N, Soejima A, Nakano K, Yamochi T, Nakashima M, Kobayashi S, Tanaka Y (2016). Polycomb-dependent epigenetic landscape in adult T-cell leukemia. Blood.

[18] Yamagishi M, Nakano K, Miyake A, Yamochi T, Kagami Y, Tsutsumi A, Matsuda Y, Sato-Otsubo A, Muto S, Utsunomiya A (2012). Polycomb-mediated loss of miR-31 activates NIK-dependent NF-κB pathway in adult T cell leukemia and other cancers. Cancer Cell.

[19] Tattermusch S, Skinner JA, Chaussabel D, Banchereau J, Berry MP, McNab FW, O'Garra A, Taylor GP, Bangham CR (2012). Systems biology approaches reveal a specific interferon-inducible signature in HTLV-1 associated myelopathy. PLoS Pathog.

[20] Vernin C, Thenoz M, Pinatel C, Gessain A, Gout O, Delfau-Larue MH, Nazaret N, Legras-Lachuer C, Wattel E, Mortreux F (2014). HTLV-1 bZIP factor HBZ promotes cell proliferation and genetic instability by activating OncomiRs. Cancer Res.

[21] Ritchie ME, Phipson B, Wu D, Hu Y, Law CW, Shi W, Smyth GK (2015). Limma powers differential expression analyses for RNA-sequencing and microarray studies. Nucleic Acids Res.

[22] Sanz H, Valim C, Vegas E, Oller JM, Reverter F (2018). SVM-RFE: selection and visualization of the most relevant features through non-linear kernels. BMC bioinformatics.

[23] Wang C, Xiao Z, Wu J (2019). Functional connectivity-based classification of autism and control using SVM-RFECV on rs-fMRI data. Physica Medica.

[24] Samb ML, Camara F, Ndiaye S, Slimani Y, Esseghir MA (2012). A novel RFE-SVM-based feature selection approach for classification. Int J Adv Sci Technol.

[25] Salih SJ, Ghobadi MZ (2022). Evaluating the cytotoxicity and pathogenicity of multi-walled carbon nanotube through weighted gene co-expression network analysis: a nanotoxicogenomics study. BMC Genomic Data.

[26] Huang H-Y, Lin Y-C-D, Li J, Huang K-Y, Shrestha S, Hong H-C, Tang Y, Chen Y-G, Jin C-N, Yu Y (2020). miRTarBase 2020: updates to the experimentally validated microRNA–target interaction database. Nucleic Acids Res.

[27] Chen J, Bardes EE, Aronow BJ, Jegga AG (2009). ToppGene suite for gene list enrichment analysis and candidate gene prioritization. Nucleic Acids Res.

[28] Malpica L, Pimentel A, Reis IM, Gotuzzo E, Lekakis L, Komanduri K, Harrington T, Barber GN, Ramos JC (2018). Epidemiology, clinical features, and outcome of HTLV-1–related ATLL in an area of prevalence in the United States. Blood Adv.

[29] Malpica L, Enriquez DJ, Castro DA, Peña C, Idrobo H, Fiad L, Prates M, Otero V, Biglione M, Altamirano M (2021). Real-world data on adult T-cell leukemia/lymphoma in Latin America: a study from the grupo de estudio latinoamericano de linfoproliferativos. JCO Global Oncol.

[30] Katsuya H, Ishitsuka K, Utsunomiya A, Hanada S, Eto T, Moriuchi Y, Saburi Y, Miyahara M, Sueoka E, Uike N (2015). Treatment and survival among 1594 patients with ATL. Blood.

[31] Kataoka K, Iwanaga M, Yasunaga JI, Nagata Y, Kitanaka A, Kameda T, Yoshimitsu M, Shiraishi Y, Sato-Otsubo A, Sanada M (2018). Prognostic relevance of integrated genetic profiling in adult T-cell leukemia/lymphoma. Blood.

[32] Kogure Y, Kameda T, Koya J, Yoshimitsu M, Nosaka K, Yasunaga JI, Imaizumi Y, Watanabe M, Saito Y, Ito Y (2022). Whole-genome landscape of adult T-cell leukemia/lymphoma. Blood.

[33] Fang X, Zhang D, Zhao W, Gao L, Wang L (2020). Dishevelled associated activator of morphogenesis (DAAM) facilitates invasion of hepatocellular carcinoma by upregulating hypoxia-inducible factor 1α (HIF-1α) expression. Med Sci Monit.

[34] Mei J, Huang Y, Hao L, Liu Y, Yan T, Qiu T, Xu R, Xu B, Xiao Z, Jiang X (2019). DAAM1-mediated migration and invasion of ovarian cancer cells are suppressed by miR-208a-5p. Pathol Res Pract.

[35] Xiong H, Yan T, Zhang W, Shi F, Jiang X, Wang X, Li S, Chen Y, Chen C, Zhu Y (2018). miR-613 inhibits cell migration and invasion by downregulating Daam1 in triple-negative breast cancer. Cell Signal.

[36] Wang H, Zhang X, Liu Y, Ni Z, Lin Y, Duan Z, Shi Y, Wang G, Li F (2016). Downregulated miR-31 level associates with poor prognosis of gastric cancer and its restoration suppresses tumor cell malignant phenotypes by inhibiting E2F2. Oncotarget.

[37] Leng A, Liu T, He Y, Li Q, Zhang G (2009). Smad4/Smad7 balance: a role of tumorigenesis in gastric cancer. Exp Mol Pathol.

[38] Zeng J, Jiang B, Xiao X, Zhang R (2020). Inhibition of sphingosine kinase 2 attenuates hypertrophic scar formation via upregulation of Smad7 in human hypertrophic scar fibroblasts. Mol Med Rep.

[39] Li R, Dong X, Ma C, Liu L (2014). Computational identification of surrogate genes for prostate cancer phases using machine learning and molecular network analysis. Theor Biol Med Model.

[40] Schiewer MJ, Goodwin JF, Han S, Brenner JC, Augello MA, Dean JL, Liu F, Planck JL, Ravindranathan P, Chinnaiyan AM (2012). Dual roles of PARP-1 promote cancer growth and progression. Cancer Discov.

[41] Zhang Z, Hildebrandt EF, Simbulan-Rosenthal CM, Anderson MG (2002). Sequence-specific binding of poly (ADP-ribose) polymerase-1 to the human T cell leukemia virus type-I tax responsive element. Virol J.

[42] Jiang P, Desai A, Ye H (2021). Progress in molecular feature of smoldering mantle cell lymphoma. Exper Hematol Oncol.

[43] Xu XM, Qian JC, Deng ZL, Cai Z, Tang T, Wang P, Zhang KH, Cai J-P (2012). Expression of miR-21, miR-31, miR-96 and miR-135b is correlated with the clinical parameters of colorectal cancer. Oncol Lett.

